# Epidemiology Pattern, Prevalent Genotype Distribution, Fighting Stigma and Control Options for Hepatitis D in Bulgaria and Other European Countries

**DOI:** 10.3390/life13051115

**Published:** 2023-04-30

**Authors:** Denitsa Todorova Tsaneva-Damyanova, Lora Hristova Georgieva

**Affiliations:** 1 Department of Microbiology and Virology, Medical University, 3 Bregalnitsa St., 9000 Varna, Bulgaria; 2 Department of Social Medicine and Healthcare Organization, Medical University, 9000 Varna, Bulgaria

**Keywords:** HBV, chronic hepatitis HDV, epidemiology, genotype, viral control

## Abstract

Hepatitis D virus (HDV) is a satellite virus that causes the most aggressive form of all viral hepatitis in individuals already infected with HBV (hepatitis B virus). In recent years, there has been a negative trend towards an increase in the prevalence of chronic hepatitis D in Europe, especially among immigrant populations coming from regions endemic for the virus. The aim of this review is to analyse the current epidemiology of chronic HDV, routes of transmission, prevalent genotype, its management, prevention, fighting stigma and options for viral control in European countries, such as Bulgaria.

## 1. Introduction

The hepatitis D virus (HDV) is a small satellite virus, the smallest yet identified in the human population, that causes the most aggressive form of all viral hepatitis strains. The history of HDV began in 1977, when Italian gastroenterologist and virologist Mario Rizzetto, from the Department of Gastroenterology in Turin, Italy, reported his discovery of a new antigen called the HBsAg-associated delta-antigen, using an immunofluorescence technique [1]. It was identified in subjects who were already infected with HBV and had severe liver disease. The official discovery of the hepatitis D virus was made in 1980 and there was a change in the nomenclature from Greek to Latin, with delta being replaced by D, as in HDV [2]. Despite increased morbidity and mortality, forty-six years after its discovery, this unique virus remains an understudied and largely underestimated enigma [3].

HDV is the only member of the genus Deltavirus, according to the International Committee on Taxonomy of Viruses (ICTV), and belongs to the Delatviridae family [4]. Recently, HDV was reclassified with other HDV-like viruses as Kolmioviridae, the only family within a new realm, Ribozyviria, where kolmio means “triangle” in Finnish, referring to the Greek letter “Δ” (delta) [5,6].

The virus genome consists of a circular single-stranded negative (–) RNA molecule of 1668–1697 ribonucleotides (depending on the genotype) [7]. HDV uses the HBsAg of HBV as an envelope and shares the same receptor for viral entry [8]. The hepatitis D virus nucleocapsid contains two isoforms of HDAg (delta-antigen particle—HDAg): large (27 kD,) and small (24 kD). HDV encodes only these two proteins. The relative ratio of these two HDAg isoforms regulates the balance between replication and viral assembly [9]. HDV does not encode for an RNA-dependent RNA polymerase, but depends on host DNA-dependent RNA polymerase for transcribing and replicating the genome into the target cell [10]. The genomic RNA of HDV is replicated by a rolling circle mechanism. Although there is a resemblance in the availability of a circular RNA genome and a mechanism of replication with viroids, HDV is clearly differentiated by its larger genome and capability to encode its protein [11].

The prevalence of HDV infection varies greatly across different geographic regions and does not exactly match the distribution of patients with chronic HBV infection [12]. Due to mandatory vaccination against HBV, HDV distribution has decreased in most European countries [3]. However, in recent years, the prevalence of chronic hepatitis D has increased in several countries, such as France, Germany and Spain, mainly among immigrant populations coming from regions endemic for the virus (Eastern Europe, Africa and Turkey) [13,14,15]. Evaluating the prevalence of HDV and its contribution towards liver dysfunction among general and specific population groups is key to guide screening, prevention, clinical care, policy enunciation, effective public health interventions and the development of new therapies [16].

There are eight different HDV genotypes (genotype 1–8) due to the obtained and aggregated data of multiple hepatitis D coding sequences and phylogenetic reconstructions. The viral genotypic diversity is related to the geographical location. As per the literature, genotype 1 dominates worldwide, as well as in Europe (89.9% of published data), while other genotypes are more localised, including genotype 2 in Asia, genotype 3 in Latin America (Amazon basin), genotype 4 in Japan and Taiwan, genotype 5 in Western Africa and genotypes 6–8 in Central Africa [16,17].

Chronic HDV infection leads to the rapid progression of liver dysfunction, increasing by a fewfold the risk of cirrhosis, decompensated cirrhosis, hepatocellular carcinoma (HCC) and the mortality rate when compared to HBV monoinfection [18]. The options for HDV viral control in European countries are focused on partial models of screening, care, prevention and dealing with discrimination, even though they do not meet the needs or improve the everyday life of patients with chronic hepatitis D [19].

This review aims to analyse the current epidemiology of chronic HDV, the routes of transmission, the prevalent genotype, its management, prevention, fighting stigma, and options for viral control in Bulgaria and other European countries.

## 2. The Contemporary Patterns of Chronic Hepatitis D: A Literature Review

### 2.1. Epidemiology of HDV in General and Hepatology Clinic Populations

Reports on the epidemiological prevalence of HDV infection are numerous, but do not provide a complete picture of its prevalence. Higher rates of HDV persist among immigrants from endemic regions. The worldwide prevalence of HDV among HBV carriers has been estimated to be around 13–15%, or a total of around 60–72 million people [20]. Other studies have reported a global HDV prevalence of around 12 million people, with anti-HDV distribution of 4.5% among HBsAg-positive individuals and 0.16% in the general population. Most researchers assume that the prevalence of dual HBV/HDV is closer to 20 million globally [21].

Like HBV, HDV can be transmitted through blood and sexual contact, but vertical transmission is rare. The target populations at higher risk of HDV infection include intravenous drug abusers (IDUs), people living with HIV and HCV, individuals with high-risk sexual behaviours and haemodialysis recipients [16].

The World Health Organization (WHO) has declared the following geographic areas as HDV endemic regions: Central and West Africa, Central and North Asia, Vietnam, Mongolia, Pakistan, Japan, Chinese Taipei, Pacific Islands (Kiribati and Nauru), the Middle East, Eastern Europe, regions of the Eastern Mediterranean, Turkey, the Amazon Basin and Greenland [22]. One meta-analysis of 62 studies based on HDV prevalence in the WHO Eastern Mediterranean region an average HDV prevalence of 15%, and up to 37% in patients with chronic hepatitis, cirrhosis, and hepatocellular carcinoma (HCC) [23]. In the literature, some authors estimate that globally, between one in five and one in six cases of cirrhosis or HCC among people with hepatitis B is due to HDV infection, indicating that hepatitis D is an important factor to liver dysfunction [16]. Interestingly, studies from the same country report discrepant hepatitis D prevalence, possibly due to significant geographic variation even within a single country’s area. For example, a meta-analysis from Turkey reports an anti-HDV seroprevalence of 4.8% in western Turkey and 46.3% in southeastern Turkey [24]. Lower rates are reported in countries that are not endemic for HDV, such as Japan, Australia and England [25]. In the USA, the prevalence of HDV among chronic HBV carriers has been reported to range from 0.36% to 2% in native citizens and up to 50% in some immigrants from endemic regions and at-risk groups [26].

In Europe, the prevalence of HDV among HBsAg-positive people was estimated at 3.0% for general population and 19.5% for patients in hepatology clinics [16]. Thanks to vaccination against HBV, compulsory testing of blood products and improvements in socio-sanitary conditions, HDV prevalence has decreased in most European countries over the last 20 years [12]. Overall, around 445,000 people in Europe are considered infected with chronic HDV [27]. The areas reporting high prevalence of chronic HDV among HBV infected people include Romania (23%) [28]; Eastern Turkey (15%) [29]; Yakutia, Siberia, Russia (18–20%) [30]; and Greenland, among Inuits (6%) [31]. Northern European countries, such as Denmark, Sweden and Norway, have low rates of chronic HBV infection, and HDV infection is only a concern for selected at-risk groups, such as IDUs [32].

To evaluate the differences in HDV prevalence across European countries, we summarized the available literature data from larger published epidemiological studies reported from 1986 to 2022 (Table 1). The materials and methods sections of these studies were carefully analysed.

According to the analysis conducted by Hayashi and colleagues, HDV is rare in Austria, Belgium, Bulgaria, the Czech Republic, Croatia, France, Greece, Ireland, Poland and Switzerland. The prevalence rates of anti-HDV have ranged from 14% to 39% in Moldova and Serbia, and from 7% to 10.29% in Albanian patients with chronic liver disease [76]. In Italy, the prevalence of anti-HDV decreased from 7.4% to 6.4% among Italians between 2001–2009, but increased from 12.9% to 26.4% in high-endemicity foci and immigrant populations [2]. The relative proportion of HDV-infected immigrants has been increasing in relation to HDV-infected natives, interrupting the decline in HDV. The prevalence has been stabilised at 8–10% in Germany, Italy, Spain and France over the last 10–15 years, where over 75% of the immigrants came from Turkey, Eastern Europe and the former Soviet Union [13,77]. This contemporary pattern of decreasing domestic and increasing migrant HDV infections has been observed in all high-income countries in Europe, presenting a challenge for healthcare systems [71].

Chronic diseases directly impact the health system by increasing the demand for health services and their associated costs. Most European healthcare systems, including Bulgaria’s, have not adapted to this major change in demand, and instead continue to be organized primarily around the active and episodic model of care, which fails to meet the needs of patients with chronic illnesses [19].

**Figure 1:**
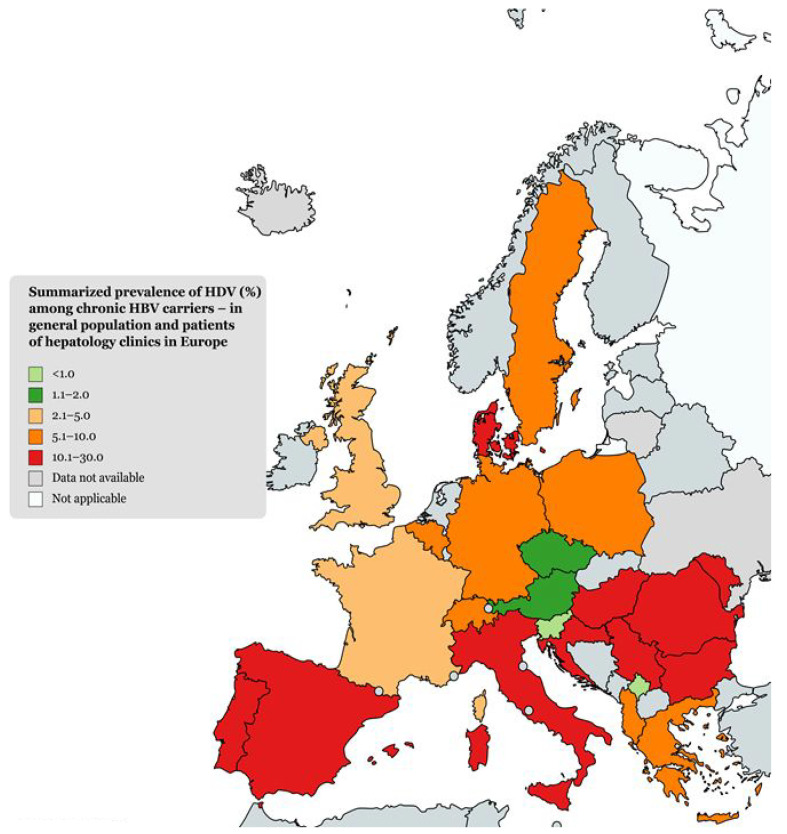
Summary prevalence of chronic HDV carriers in Europe.

We have compiled the available literature data published from larger European epidemiological studies between 1986 and 2022 to create a world map of chronic hepatitis B and D carriers (Figure 1).

In Europe, HDV infection is maintained by two different remaining pockets of HDV-infected individuals: young people who immigrate from less-developed areas where HDV is endemic, and a domestic pool of older individuals who represent the tail of an infection acquired decades ago during the HDV endemic period [12].

The epidemiological data on chronic HDV viral infection in Bulgaria is based on a few studies in the literature, performed with HDV diagnostic tests with different analytical characteristics. In 1986, Naoumov and colleagues reported the prevalence of delta infection among 105 HBsAg-positive patients with chronic liver diseases and 42 patients who had died due to fulminant hepatitis B. Delta infection was detected in 8.6% of subjects with chronic HBV infection and 7.1% of patients with fulminant hepatitis [42]. During the period of 1986–1997, Krastev and colleagues found the rate of HDV infection to be 16.1% higher among patients in hepatology clinics, with a high concentration of patients with chronic HBV and HDV infection [44]. In 1998, B. Iliev and colleagues reported that of 1465 HBsAg-positive serum samples, 151 (10.3%) were positive for delta infection. The highest percentage of infected heamophiliacs (47.06%) was followed by female prostitutes, patients with acute and chronic hepatitis and polytransfused patients [43]. A study among 1280 HBsAg (+) patients who received antiviral therapy from the National Health Insurance Fund between 2008 and 2013 showed a 3.8% rate of HDV [44]. During the period of 2013–2018, we conducted a sero-epidemiological study among 391 patients with chronic liver diseases at the “St. Marina” University Hospital in Varna city. We found 16.6% (n = 65) of them to be anti-HDV positive. We found HDV RNA positive results in 96.9% of all the anti-HDV Ab (+) patients, which was close to the results reported by EASL, according to which 87% of anti-HDV positive serum samples are also hepatitis D nucleic acid positive [45,77,78]. A study carried out for 788 inmates in five Bulgarian prisons during the period 2018–2019 showed an overall rate of antibody positivity for anti-HDV to be 10.86% (n = 84). This study showed a higher prevalence of blood-borne infections among prison inmates in comparison with the general population in Bulgaria, suggesting their probable transmission in prisons was due to intravenous drug use, unsafe sexual behaviour and tattoos [46]. All this data clearly suggests that the prevalence of HDV infection in Bulgaria is close to the average evaluation for chronic HBV/HDV carriers in Europe reported in a recent meta-analysis made by Stockdale and colleagues [16]. Nevertheless, obtaining accurate estimates regarding the epidemiology of HDV is still challenging, and careful assessment of potential biases of the representativeness of the conducted studies is needed. Heterogeneity in HDV prevalence can be expected due to variable and potentially evolving epidemic patterns in European countries, including Bulgaria, as well as non-standardized screening practices and application of HDV diagnostic tests with different analytical characteristics [79].

### 2.2. Forms of HBV/HDV Infection

There are two evident patterns of infection for HDV: co-infection and superinfection [80].

Co-infection involves simultaneous infection with HBV and HDV, and usually persists as acute hepatitis with elevated transaminases (alanine aminotransferase (ALT), aspartate aminotransferase (AST)), serum bilirubin and a higher risk of fulminant hepatitis compared to HBV monoinfection [81,82]. Acute hepatitis D occurs after an incubation period of 1–2 months, and during the preicteric phase, there are nonspecific symptoms, such as fatigue, lethargy, nausea and vomiting. The crucial diagnostic marker for acute HDV coinfection is represented by high titres of anti-HBcIgM and anti-HBc antibodies, which disappear along with the resolution of clinical symptoms. Anti-HDV IgM antibodies are not specific to acute hepatitis D, and anti-HDV IgG are low-titred and usually appear after clinical presentation [83]. Several outbreaks of very severe acute HBV/HDV hepatitis have been described in different regions of the world. In recent years, the incidence of acute HDV infection in Europe has decreased due to the successful implementation of HBV vaccination programs. A number of studies indicate that, compared to HBV monoinfection, HBV/HDV co-infection is usually transient and self-limited, and the disease becomes chronic HDV in about 10% of the cases [77]. In case of chronification, a more severe clinical course is frequent, and two peaks of serum ALT and AST may be observed [83]. HDV might also affect ALT normalization in chronic HBV carriers. When compared to chronic HBV patients without HDV infection, those with HDV coinfection had an increased risk (30-fold and 10-fold) of ALT abnormality after one and two years of therapy, respectively. Normalization of the levels of ALT during treatment was also an important indicator for long-term outcomes for these patients [84].

HBV/HDV superinfection develops in those with a history of a previous chronic HBV infection. In the case of superinfection, the preceding HBV viremia provides a biological basis for full expression of HDV virulence and pathogenicity, which can be clinically expressed as severe acute and/or fulminant hepatitis. This condition can present as an exacerbation of HBV monoinfection or as newly diagnosed hepatitis in a previously asymptomatic HBsAg carrier [85]. The levels of ALT and AST are persistently elevated in most patients. In chronic hepatitis D, high titres of HDAg and anti-HDV antibodies are typically found. According to literature data, HDV-superinfection of a chronically HBV-infected individual usually causes more severe acute hepatitis with a shorter incubation period and leads to chronification in more than 90% of cases [79].

HDV cannot replicate successfully into the target hepatocytes until HBV has infected a sufficient number of cells; therefore, HBV infection is a limiting factor for HDV infection. The hepatitis D virus usually causes suppression of HBV replication in approximately 70% of cases, with HBV–HDV co-dominance found in 28% of cases and, less commonly, HBV-dominant cases at 3% [86]. In the course of HDV superinfection, the serum level of HDV RNA can reach 10^12^ copies/mL within a few weeks from the time of infection [83].

Because of the differences in prognosis and treatment, discriminating between acute HBV/HDV co-infection and superinfection in these patients is critical [8,87]. This highlights the importance and significance of HDV screening and testing among chronic HBV carriers, especially in high endemic pockets of HDV-infected individuals from Eastern Europe, Asia and Africa [88].

According to published data, 26% of patients with chronic hepatitis B monoinfection develop liver cirrhosis with a permanent disability and lower survival rate, and 10% of them develop hepatocellular carcinoma [86]. Longitudinal studies have confirmed that 80% of chronic hepatitis D carriers develop cirrhosis, which is significantly higher than the percentage seen in HBV monoinfected patients [89]. On average, HDV infection progresses to cirrhosis within 5 years and to HCC within 10 years. HDV was responsible for almost half of the liver cirrhosis and HCC cases in Turkey [24]. A few studies conducted in Italy, Spain, Greece and Germany confirmed that the more severe course in HDV superinfection, and the faster the progress to liver dysfunction [15]. A study conducted in Romania among 166 patients with HDV-related cirrhosis showed that 12% of them had already developed HCC [28]. One research study conducted in the period of 2016–2020 among 36 patients with chronic HBV/HDV superinfection in Varna, Bulgaria showed that 75.0% were already diagnosed with cirrhosis, 13.9% were diagnosed with HCC and 16.7% died within the 5-year study period [78].

### 2.3. Prevalent HDV Genotypes and Their Pathogenicity

Due to the sequence variations found in the HDV isolates, eight clades-termed HDV genotypes have been documented in the human population (HDV-1 to HDV-8) [7]. The HDV genotypes differ in their genomic sequence by 19–40% and are sub-divided into 2 to 4 subgenotypes, except HDV-3 [11]. In accordance with current virus taxonomy in genus Deltavirus, these eight HDV genotypes are designated as different species: italiense (HDV-1), japanense (HDV-2), peruense (HDV-3), taiwanense (HDV-4), togense (HDV-5), careens (HDV-6), cameroonense (HDV-7) and senegalense (HDV-8) [6].

The viral genotype diversity is related to the geographical location. Isolates of HDV genotype 1 are found throughout the world (89.9% of published data) and have a variable course of infection, ranging from asymptomatic infection to fulminant hepatitis. HDV-genotype 1 is present in Europe, North America, South Asia, Eastern Mediterranean and the Middle East [90]. HDV genotype 2 is found mostly in Asia, including Japan, Taiwan, and, recently, in Yakutia (Russia) [30]. HDV-genotype 2 is associated with a higher rate of remission than italiense [91]. HDV-genotype 3 is isolated only in the northern parts of South America (Peru, Venezuela, Colombia) and is associated with the most severe and aggressive forms of hepatitis D [92]. Genotype 4 occurs in Japan and Taiwan and has a heterogeneous pathogenesis, leading to milder forms of liver dysfunction. However, genotype-4 isolated from Okinawa, Japan is associated with a faster progression to cirrhosis, compared to the predominant genotype 4 in Taiwan [93]. Patients chronically infected with HDV-1 and HDV-3 experience more severe hepatitis than those infected with genotypes HDV-2 and HDV-4 [20]. HDV-genotypes 5 to 8 are found in patients from Africa who migrated to Northern Europe, and a natural history of the infection is still not well characterized [94]. European HDV-1 and African HDV-5 patients at higher risk of developing cirrhosis [17]. All the genotypes of HDV refer to one viral serotype [47].

In Bulgaria, HDV genotyping has only been analysed in a few studies, and HDV-genotype 1 was found to be prevalent, similar to the other neighbouring countries [44]. We conducted an investigation among 12 Bulgarian chronic HDV patients, aged 28 to 62 years, during the period of 2013–2019. All the patients were genetically analysed via direct sequencing of the HDV RNA amplicons. The genotype assignment was based on the analysis of the sequences that corresponded to nucleotides between 906 and 1256. When comparing the obtained HDV sequences with sequences correlating to HDV from the BLAST (basic local alignment search tool) database in NCBI (National Center for Biotechnology and Information), they were all closely related to HDV-genotype 1 [95,96].

### 2.4. Management of Chronic Hepatitis D and Options for Viral Control

#### 2.4.1. HDV Laboratory Tests

##### Anti-HDV Antibody (Ab) IgM and IgG

An essential challenge in HDV diagnostic testing is the validity, standardization and comparability of HDV assays [16]. Among the people with an HBV infection, testing for HDV markers can be performed via immunoenzymatic (EIA), molecular and electron microscopic methods. In everyday laboratory and clinical practice, serological methods are the main testing procedure—antibody detection by enzyme-linked immunosorbent assay (ELISA). In the case of a simultaneous HBV/HDV infection, there are positive results for HDV Ab class IgG and IgM and HDV RNA (with commercially available kits, such as Dia pro, Adaltis, DiaSorin, etc.) [97]. Anti-HDV Ig M is detectable for 2–3 weeks from the beginning of the infection and disappears after 2 months in patients with acute HDV. Anti-HDV Ig M persists longer in chronic HDV patients [98].

##### HDV Antigen (Ag)

In order to detect HDV Ag, a liver biopsy must be performed, followed by measurement using immunohistochemistry. The contemporary usage of molecular techniques has drastically reduced the use of immunohistochemistry. In general, immunohistochemistry methods are not used routinely, as there are no commercially available kits for liver HDVAg determination [99]. A few studies reported that in immunocompetent individuals, HDV-Ag is frequently neutralized by anti-HDV antibodies and is not detectable. In contrast, HDV- Ag is usually detected in serum samples received from immunocompromised patients and chronic HDV carriers [100].

##### HDV RNA

While various methods of detection of anti-HDV-IgG are commonly used, the results from EIA testing can be confirmed with polymerase chain reaction (PCR) via HDV RNA detection. HDV PCR shows positive results in all of the chronic HDV carriers. The hepatitis D viral load can be defined via qualitative and quantitative PCR (with commercially available kits -Roche diagnostics, Primerdesign, etc.) [99]. Although it is not widely available and not yet fully standardized [27], the first standardized test for HDV RNA was created by WHO in 2013 [22]. This test is used to monitor the viral load and predict the antiviral treatment response [91].

The serological markers for HBV (HBsAg), HDV (anti-HDV total) and HDV RNA (+), as well as histological data for HDV for more than 6 months define chronic hepatitis D [101]. The anti-HDV Ab is a marker of exposure to HDV, and its clinical interpretation must be correlated with the medical status of the HBsAg carrier recruited for the examination [78]. In asymptomatic HBsAg carriers at low risk of HDV, the HDV Ab most often represents the serological scar of a past resolved infection [2].

#### 2.4.2. HDV Awareness and Screening Guidelines

**Figure 2:**
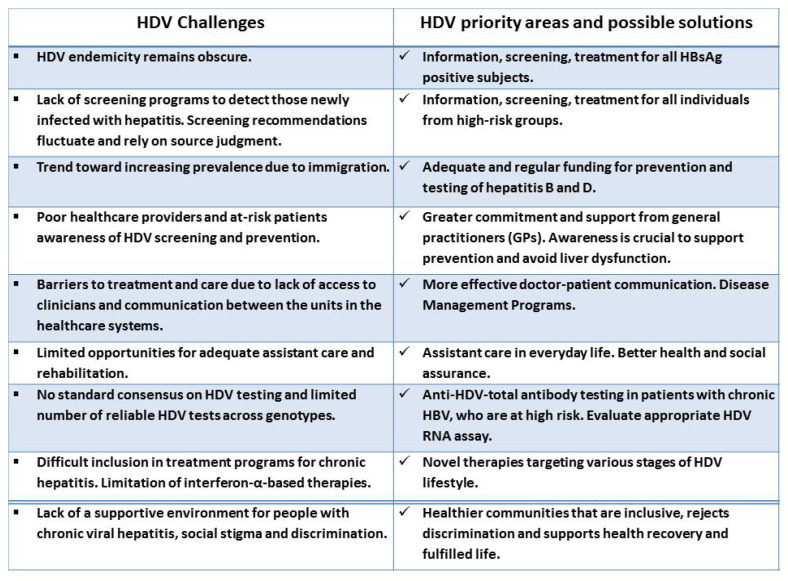
HDV challenges, priority areas and possible solutions.

The key recommendations of different societies currently differ in the screening strategy for HDV diagnosis, and still, there is no standard consensus on the screening and testing of chronic HDV worldwide [102]. The major challenges that hamper HDV management, as well as key priority areas and possible solutions, are presented in Figure 2.

In the United States, the American Association for the Study of Liver Disease (AASL) suggests that total antibody tests should be performed in all HBsAg-positive persons at risk for HDV, including those with HIV infection, persons who inject drugs, men who have sex with men, those at risk for sexually transmitted diseases and immigrants from areas of high HDV endemicity [27]. However, there are no peer-reviewed references that suggest risk-based testing will identify all HDV-infected individuals or that this testing approach will lead to HDV eradication [100]. However, given the severity of diseases caused by HDV, a recent trend in the data suggesting a higher-than-expected prevalence, and increasing evidence point to suboptimal diagnosis of HDV infection. The European Association for the Study of the Liver (EASL), as well as the Asian Pacific Association for the Study of the Liver (APASL) recommend universal testing for HDV in anyone with chronic hepatitis B [78,102]. These chronic HBV carriers can be immigrants from HDV endemic areas with already-diagnosed liver dysfunction or with abnormally high liver enzymes during HBV treatment [103]. EASL and APASL recommend screening for HDV to be performed by ELISA anti-HDV IgG, and, if positive, HDV PCR should be performed [98]. As per the World Gastroenterology Organisation (WGO), anti-HDV screening is recommended, particularly if hepatitis is present in the face of little or no HBV viral replication or HBsAg (+) people from endemic HDV regions and IDUs [27].

The enhancement of screening coverage was targeted as a major WHO strategy to meet their goal of eliminating viral hepatitis by 2030. This strategy estimates the global burden of disease from viral hepatitis, and it has been defined as a major goal in the first global health sector strategy on viral hepatitis from 2016–2021, in the context of the European region. The strategy addresses all five hepatitis viruses (hepatitis A, B, C, D and E), with a particular focus on hepatitis B and C, owing to the relative public health burden they represent. The goal of the global health strategy is the elimination of viral hepatitis as a public health threat in the WHO European region by 2030 through the reduction of transmission, morbidity and mortality due to viral hepatitis and its complications, and by ensuring equitable access to comprehensive prevention, recommended testing, care and treatment services for all [104].

Disruption of viral hepatitis screening and diagnosis programs caused by the COVID-19 pandemic has slowed progress toward the stated global goals. Screening of all individuals with chronic hepatitis B would not only enable a more accurate determination of the HDV prevalence, but would also lead to on-time therapeutic interventions, reducing the burden of chronic HDV complications and improving access to medical care [105].

In Bulgaria, there is no national program for screening, prevention, control and management of viral hepatitis. In 2021, the National Program for Prevention and Control of Viral Hepatitis in the Republic of Bulgaria 2021–2025 was introduced [106]. The lack of a national vision for chronic disease control with nationally integrated care strategies has led to the lack of a supportive environment for people with chronic viral hepatitis D, their partners and relatives, who bear the burden of social stigma and discrimination. Patients suffering from this insidious disease were deprived of the opportunity to proactively seek information from a reliable source, a clearly defined course of action, adequate treatment, control and rehabilitation. In 2021, the National Program for Prevention and Control of Viral Hepatitis in the Republic of Bulgaria 2021–2025 was introduced [105]. With the support of the National Program in 2022, on World Hepatitis Day, the National Center for Infectious and Parasitic Diseases (NCIPD) in Bulgaria joined the Bulgarian Ministry of Health’s initiative for free hepatitis screening testing in Bulgaria [107].

#### 2.4.3. Management of Chronic HDV

The treatment of HDV infection has not changed a lot in the last 25–30 years and is based on WHO recommendations for treatment with pegylated interferon б and pegylated interferon б (PegIFN б-since 2005) for 48 weeks; however, this has limited effects [27]. Combining therapy with nucleos(t)ide analogues (Lamivudine, Adefovir, Tenofovir) have not improved the overall outcomes [82].

**Figure 3:**
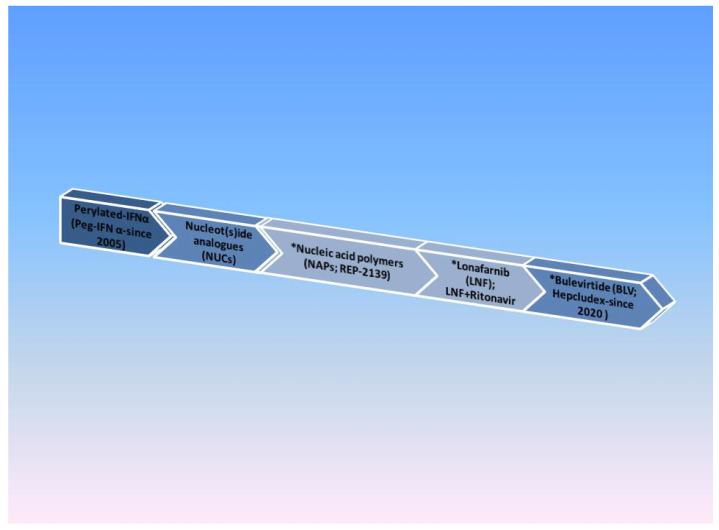
HDV drug timeline. Note: * Current therapies under evaluation.

The current therapeutic strategies rely on preventing HBsAg particles from assembling, thus preventing HDV export into the bloodstream, interfering with l-HD-Ag farnesylation in order to block HDV assembly. Other strategies are associated with HBsAg binding to NTCP (sodium taurocholate co-transporting polypeptide) to prevent HDV entering hepatocytes [2]. The contemporary treatment of chronic HDV can be seen in Figure 3.

##### Nucleic Acid Polymers (NAPs)

Nucleic acid polymers, such as REP2139, can cause a significant reduction in HBsAg levels in HDV chronic carriers by inhibiting the synthesis of HBsAg subviral particles. A phase 2 trial that combined REP2139 with PegIFNб showed encouraging preliminary results in 12 patients with chronic HDV [108].

##### The Farnesyl-Transferase Inhibitor Lonafarnib (LNF)

Lonafarnib is a prenylation inhibitor that inhibits HDV virion assembly. In a phase 2 double-blinded trial, LNF significantly reduced viremia [27]. Lonafarnib combined with Ritonavir (a protease inhibitor) sustained a good antiviral response for 24 weeks of therapy [109].

##### Bulevirtide (BLV; Hepcludex)

Bulevirtide (previously known as Myrcludex B) is a hepatocyte inhibitor of HDV entry. It decreases the levels of HDV-infected hepatocytes and allows recolonization with HDV-free regenerating cells [110]. In 2020, the first antiviral agent for HDV was approved by the European Medical Agency under the trade name Hepcludex. It is considered for patients with compensated liver dysfunction and HDV RNA positive results [111].

#### 2.4.4. Life with Chronic HDV—Fighting Stigma and Discrimination

In many European countries, people living with infectious bloodborne viruses, such as hepatitis B virus and hepatitis D, frequently face stigma and discrimination in their everyday life. Despite a high global prevalence of the disease, stigma related to HBV is less well-characterized, which may be partly attributable to a lack of settled political priorities around HBV [112]. The stigma directed towards people living with HBV and HDV arises from multiple sources. These include prejudices that this person may live in poor sanitary conditions, may be IDU or may have had lots of sexual partners, as well as an irrational fear of infection, often powered by a lack of awareness and understanding of routes for transmission of HBV and HDV [113]. One study from Europe assessed the attitudes towards HBV in Turkish immigrants to the Netherlands, as stigma around HBV/HDV is best characterized in literature in a Chinese setting [112].

A study from Bulgaria was identified in the current review, and this study assessed stigma towards 30 patients with chronic HDV conducted in Varna, Bulgaria in 2021. When the participants were asked if they had been discriminated against because of their chronic suffering, 90% of the respondents answered positively. The most frequently reported areas of life of chronic hepatitis D patients reported in the survey and marked by stigma were discrimination from society (67.0%), healthcare professionals (18.5%) and institutions (14.5%) [78,114]. The reported results coincided with those reported by the World Hepatitis Alliance, where more than 90% of surveyed patients in different countries reported stigma and discrimination: 53% of respondents are socially isolated; 1 in 2 people were restricted from receiving quality healthcare; and 42% lost their job or income. From social exclusion and unequal healthcare, to limited employment opportunities and verbal attacks, stigma and discrimination prevent people living with viral hepatitis from leading normal lives and fulfilling their potential. More national initiatives are needed in European countries, including Bulgaria, to document and combat stigma, as well as its clinical and socioeconomic consequences [115].

## 3. Conclusions

Hepatitis D is more common than expected among people with chronic HBV in Europe, and the liver health of high-risk groups and already infected individuals depends on effective viral control. Although an important factor in human pathology, accurate estimates of HDV epidemiology, screening, prevention and management are challenging due to data gaps and the lack of a consistent public health testing approach and successful treatment options. This information is crucial to guide clinical care, policy formulation and the development of new medicines in line with the global aim of hepatitis elimination by 2030. Patients with HDV experience more rapid progression and decompensation of liver disease compared to HBV monoinfection. HDV leads to a number of negative consequences in the daily life of HDV chronical carriers, from difficulties in performing daily duties at home and at work, to fighting stigma and discrimination. These facts define the necessity to increase the awareness of this disease and create a favorable public environment for changing the temporal trends and reduce the risk of infection with viral hepatitis D, as well as screening, novel therapies and HBV vaccination.

## Figures and Tables

**Table 1:** Prevalence of hepatitis D virus among HBsAg-positive carriers in European countries, based on data published from 1986 to 2022.

Country	HDV Prevalence among HBsAg-Positive Carriers (*%*)	Tested Population (Years of Research)	Reference
**Italy**	494/2001 (24.7%) 364/1556 (23.4%) 143/996 (14.4%) 69/834 (8.3%) 112/1386 (8.1%) Native: 1/78 (1.3%); Immigrants: 6/76 (7.9%) Native: 19/381 (5.0%); Immigrants: 5/107 (4.7%) Native: 53/716 (7.4%); Immigrants: 34/295 (11.5%) 78/786 (9.9%) Native: (6.4%); Immigrants: (26.4%)	HBV-infected patients (1978–1981) Chronic HBsAg carriers (1987) Chronic HBsAg carriers (1992) HBsAg carriers (1997) Chronic HBsAg carriers (2006–2007) Chronic HBsAg carriers in Ferrara (1997–2009) Chronic HBsAg carriers in Milan (2007–2008) Chronic HBsAg carriers Chronic HBsAg carriers (2019)	Smedile et al., 1983 [33] Sagnelli et al., 1992 [34] Sagnelli et al., 1997 [35] Gaeta et al., 2000 [36] Stroffolini et al., 2009 [37] Contini et al., 2012 [38] De Paschale et al., 2012 [39] Brancaccio et al., 2014 [40] Stroffolini et al., 2020 [41]
**Bulgaria**	9/105 (8.6%) 151/1465 (10.3%, of which 47.06% were haemophiliacs) 28/173 (16.1%) (49/1280) (3.8%) 65/391 (16.6%) 84/788 (10.6%)	Chronic HBsAg carriers (HDAg) (1985–1986) Chronic HBsAg carriers (1997–1998) Patients with chronic HBsAg (1986–1997) Chronic HBsAg carriers on antiviral therapy (2008–2013) Chronic HBsAg patients with liver dysfunction (2013–2018) Inmates in 5 prisons (2018–2019)	Naoumov et al., 1986 [42] Iliev et al., 2001 [43] Krastev et al., 2013 [44] Tsaneva-Damyanova, 2019 [45] Popov, et al., 2020 [46]
**Greece**	1997–2010: 90/2137 (4.2%): - Native (2.8%) - Immigrants (7.5%) - Children (15.3%)	Chronic HBsAg carriers (1997–2010)	Manesis et al., 2013 [47]
**Germany**	258/2083 (8.0%) (1992); (10.9%) (2006) 266/2354 (11.3%) 210/2844 (7.4%)	HDV infected patients and chronic HBsAg carriers (1992–2006) Chronic HBsAg carriers in Hannover (1992–2006) Chronic HBsAg carriers in Frankfurt (2000–2011)	Heidrich B et al., 2009 [48] Wedemeyer et al., 2007 [13] Rehnheimer et al., 2012 [14]
**France**	89/4492 (2.0%); (1997–2011) 1997–2005: 33/2831 (1.2%) 2010: 13/200 (6.5%) 2011: 2/234 (0.9%)	HBsAg-positive blood donors (1997–2011) HDV-Ab; HDV RNA	Servant-Delmas et al., 2014 [15]
**United Kingdom**	9/401 (2.2%) 82/962 (8.5%) (2.6%) (2000) 22/1048 (2.1%) 162/3610 (4.5%)	Chronic HBsAg carriers in Northern Ireland (1970–1989) Chronic HBV patients (mostly immigrants) (2000–2006) in London Chronic HBsAg carriers in London HBsAg carriers (2008–2012) in London (anti-HDV Ab, anti-HDV IgM, HDV RNA) HBsAg carriers (mostly immigrants) (2005–2012) in London	Curran et al., 1991 [49] Cross et al., 2008 [50] Stockdale, et al., 2020 [16] William Tong et al., 2013 [51] El Bouzidi et al., 2015 [52]
**Austria**	4/138 (2.9%) (N/A) (0.8%)	HBsAg carriers (N/A) HBV patients (N/A)	Frisch-Niggemeyer and Kunz, 1985 [53] Jachs M et al., 2021 [54]
**Albania**	1995: 10/106 (9.4%); 2005: 7/99 (7.1%)	Patients with chronic viral and/or alcohol-induced liver disease (1995 and 2005)	Kondili et al., 2010 [55]
**Slovenia**	3/1305 (0.23%)	Patients with chronic HBV infection (1998–2015)	Jelen et al., 2016 [12]
**Belgium**	44/800 (5.5%)	Chronic HBsAg carriers (2008–2009)	Ho et al., 2013 [56]
**Denmark**	29/100 (29.0%)	Chronic HBV patients (1970–1985)	Krogsgaard et al., 1988 [57]
**Croatia**	19/100 (19.0%)	Chronic HBsAg carriers (N/A)	Jelić and Jelić, 1994 [58]
**Hungary**	16/118 (13.6%)	Chronic HBsAg carriers (N/A)	Horváth et al., 1992–1993 [59]
**Chech Republic**	3/170 (2.0%)	Patients with coinfection HBV + chronic hepatitis D (2011–2020)	Hříbek, et al., 2022 [60]
**Moldova**	27/148 (18.5%)	Patients with primary liver malignancies	Turcanu et al., 2019 [61]
**Kosovo**	1/1287 (0.08%)	General population included in routine blood testing	Quaglio et al., 2008 [62]
**Serbia and** **Montenegro**	69/614 (11.2%)	Chronic HBsAg carriers (N/A)	Delić et al., 1993 [63]
**Poland**	4/102 (3.9%) 3/63 (4.8%) 5/63 (7.9%)	Chronic HBV patients (N/A) Chronic HBsAg carriers (2002–2004) (anti-HDV Ab, HDV RNA)	Chlabicz et al., 2003 [64] Bielawski et al., 2006 [65]
**Romania**	223/1094 (20.4%) 617/2761 (23.1%)	Chronic HBsAg carriers (2005) Chronic HBsAg carriers (N/A)	Popescu et al., 2013 [66] Gheorghe et al., 2015 [28]
**Portugal**	N/A (17.3%)	Chronic HBsAg carriers (N/A)	Ramalho et al., 1987 [67]
**Spain**	249/1220 (20.4%) 17/1147 (1.5%) 1984/2518 (78.8%) 100/1215 (8.2%) N/A (30%—1990s to 4.2%—2018)	Immigrants (HBsAg carriers) from Equatorial Guinea (2002–2008) HIV-positive patients (2004) African immigrants (HBsAg carriers) Chronic hepatitis B virus (HBV) patients (1983–2012) Anti-HDV Ab among active HBsAg-positive IVDUs (1990–2018)	Rivas et al., 2013 [68] Fernández-Montero et al., 2014 [69] Cuenza-Gómez et al., 2016 [70] Ordieres et al., 2017 [71] Aguilera et al., 2018 [72]
**Sweden**	N/A 650/9160 (7.1%)	Chronic HBsAg carriers (1997–2008)	Ji et al., 2012 [73]
**Switzerland**	101/1699 (5.9%) 15/338 (4.4%)	Chronic HBV patients (mostly immigrants) (N/A)-HDV Ab, HDV Ag, HDV RNA HBsAg carriers (2002–2013)	Genné and Rossi, 2011 [74] Hirzel et al., 2015 [75]

Abbreviations: HbsAg—surface antigen of the hepatitis B virus; anti-HDV Ab—anti-HDV antibodies; HDV Ag—hepatitis D antigen; IVDUs—intravenous drug abusers; N/A—not available.

## Data Availability

Not applicable.
